# UAVs and Machine Learning Revolutionising Invasive Grass and Vegetation Surveys in Remote Arid Lands

**DOI:** 10.3390/s18020605

**Published:** 2018-02-16

**Authors:** Juan Sandino, Felipe Gonzalez, Kerrie Mengersen, Kevin J. Gaston

**Affiliations:** 1 Institute for Future Environments; Robotics and Autonomous Systems, Queensland University of Technology (QUT), 2 George St, Brisbane City QLD 4000, Australia; felipe.gonzalez@qut.edu.au; 2 School of Mathematical Sciences; ARC Centre of Excellence for Mathematical & Statistical Frontiers (ACEMS), Queensland University of Technology (QUT), 2 George St, Brisbane City QLD 4000, Australia; k.mengersen@qut.edu.au; 3 Environment and Sustainability Institute, University of Exeter, Penryn, Cornwall TR10 9FE, UK; K.J.Gaston@exeter.ac.uk

**Keywords:** biosecurity, buffel grass, *Cenchrus ciliaris*, drones, remote surveillance, spinifex, *Triodia* sp., unmanned aerial vehicles (UAV), vegetation assessments, xgboost

## Abstract

The monitoring of invasive grasses and vegetation in remote areas is challenging, costly, and on the ground sometimes dangerous. Satellite and manned aircraft surveys can assist but their use may be limited due to the ground sampling resolution or cloud cover. Straightforward and accurate surveillance methods are needed to quantify rates of grass invasion, offer appropriate vegetation tracking reports, and apply optimal control methods. This paper presents a pipeline process to detect and generate a pixel-wise segmentation of invasive grasses, using buffel grass (*Cenchrus ciliaris*) and spinifex (*Triodia* sp.) as examples. The process integrates unmanned aerial vehicles (UAVs) also commonly known as drones, high-resolution red, green, blue colour model (RGB) cameras, and a data processing approach based on machine learning algorithms. The methods are illustrated with data acquired in Cape Range National Park, Western Australia (WA), Australia, orthorectified in Agisoft Photoscan Pro, and processed in Python programming language, scikit-learn, and eXtreme Gradient Boosting (XGBoost) libraries. In total, 342,626 samples were extracted from the obtained data set and labelled into six classes. Segmentation results provided an individual detection rate of 97% for buffel grass and 96% for spinifex, with a global multiclass pixel-wise detection rate of 97%. Obtained results were robust against illumination changes, object rotation, occlusion, background cluttering, and floral density variation.

## 1. Introduction

Over recent decades, invasive grasses have resulted in very substantial losses to native ecosystems around the world. Governmental, scientific, and community efforts to monitor and control these and other introduced plant species have been extremely challenging due to restricted and difficult access to remote areas, expensive operational costs, and, in some cases, hazardous data collection campaigns [1]. In Australia, the U.S., South Africa, and other parts of the world, introduced grasses have flourished in arid landscapes due to their tenacity under hot, heavy grazing, and drought conditions [2,3]. Moreover, they have been fostered by farmers because of the economic benefits that they bring through land rehabilitation and livestock production. However, many of these plant species have invaded some of the wetter and more fertile parts of the landscape and affected the survival of native plant and animal populations [4,5,6]. As a result, these species have now been catalogued as invasive plants or weeds. There is an increasing body of research focused on assessing the biodiversity effects of invasive grasses, which shows that their expansion rates are likely to exceed new high records due to climate change effects [6,7,8,9,10,11]. Straightforward, efficient, and accurate surveillance methods are required to quantify expansion rates of invasive grasses and apply reliable and efficient control methods.

Among the present efforts to monitor invasive grasses and other vegetation, different investigations have developed diverse solutions, using various image sensors and detection methods to meet a range of needs. Previously, satellite and manned aircraft imagery was used to map invasive grass infestations. Nonetheless, advances in unmanned aerial vehicles (UAV) design and path planning [12,13] have seen an increased application of remote sensing for ecological assessments and biosecurity applications [14,15,16,17]. Research from Olsson et al. [18], for instance, demonstrated the importance of using hyperspectral imagery for invasive grass detection as compared with satellite imagery. A feasibility study of sensing technology by Marshall et al. [19], for example, illustrates the potential for regional mapping of buffel grass infestations in arid landscapes using high-resolution aerial photography in red, green, blue (RGB) colour model at a cm/pixel scale over multi- and hyperspectral technologies for overall detection rates.

Weed mapping using different image sensors capable of sensing multiple spectral bands is also an active field of research. Alexandridis et al. [20], for instance, developed an approach by integrating UAVs and multispectral imagery for weed mapping, achieving detection rates of up to 96%. Moreover, Blaschke et al. [21] and Torres-Sánchez et al. [22] showed the use of Geographic Object-Based Image Analysis (GEOBIA) through UAVs and multispectral imagery to obtain detection rates of approximately 90%.

Development of image and data processing techniques for vegetation assessments is also increasing. Amongst the popular methods, the use of spectral indexes for weed detection has gained considerable popularity, as explored by Ashourloo et al. [23], Robinson et al. [24], and Lin et al. [25]. In these cases, both supervised and unsupervised segmentation algorithms were greatly influenced by image quality, spectral bands, and ground sampling distance (GSD), among other complex considerations of the scene. In sum, a universal criterion has not yet been defined for choosing a feasible sensing technology and data processing pipeline that meets every application need [26]. This paper proposes the creation of a global approach for the surveillance of invasive grasses and related biosecurity applications by developing an automatic surveillance solution integrating UAV technology with high-resolution RGB cameras and a machine learning-based classification algorithm to process and segment the data. The presented pipeline process is illustrated with the automatic detection of buffel grass (*Cenchrus ciliaris*) and spinifex (*Triodia* sp.) in arid and semi-arid ecosystems in Australia.

## 2. Materials and Methods

### 2.1. Process Pipeline

**Figure 1:**
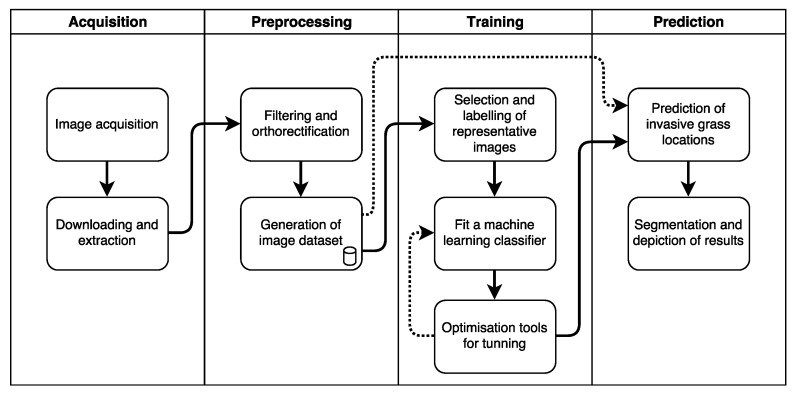
Primary pipeline for mapping of invasive grasses and related vegetation.

We developed a pipeline process consisting of four main components: Acquisition, Preprocessing, Training, and Prediction, as illustrated in Figure 1. High-resolution digital images are initially captured from a UAV flight mission. Images are downloaded, orthorectified, and preprocessed in order to extract samples with key features and label them subsequently. Data are then fed into a supervised machine learning classifier to train and optimise its detection capabilities. Finally, the entire orthorectified imagery is processed to predict the location of invasive grasses and vegetation in the studied area.

### 2.2. Site

The study site is located in the Cape Range National Park, Western Australia (WA), Australia (-22.190429, 113.865478). The site contains buffel grass, spinifex, remains of dry and decomposed vegetation, bushes, and arid soil. Images were taken in a successive series of four flight campaigns, conducted on the 10 July 2016, from 12:20 p.m. until 2:20 p.m. Meteorological conditions for that day were sunny, with south-easterly winds from 17 to 26 km/h, 46% relative humidity, 21.2 ${}^{\circ }$C mean temperature, and no precipitation [27].

**Figure 2:**
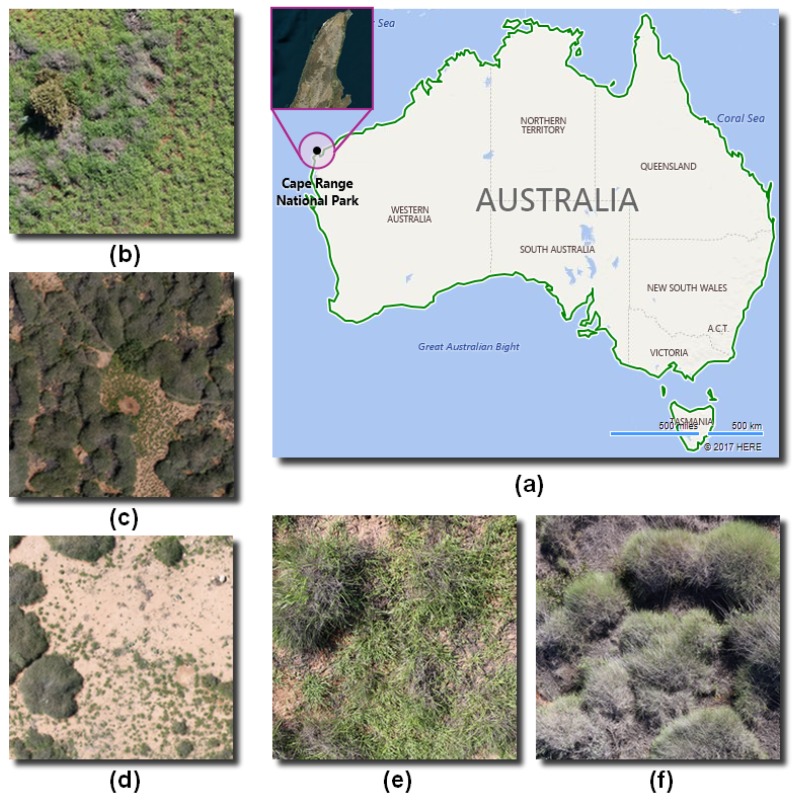
Main features of the study site. (**a**) Geographical location. (**b**) Area with high density of buffel grass. (**c**) Area with high density of spinifex. (**d**) Area with low density of invasive grasses. (**e**) Buffel grass. (**f**) Spinifex.

In the site, invasive grass species such as buffel grass and spinifex were found with negligible size variation, viewpoint variation, background clutter, and occlusion. However, they occurred at various densities, as shown in Figure 2.

### 2.3. Image Sensors

A Canon EOS 5DsR digital camera (Canon Inc., Tokyo, Japan) was utilised to capture high-resolution images. The camera specifications include 50.6 MP resolution, 28 mm focal length, ISO-400 speed, a full-frame complementary metal–oxide–semiconductor (CMOS) sensor of 36 mm × 24 mm, a 625 μs exposure time, and a global positioning system (GPS) sensor.

### 2.4. The UAV and Sample Acquisition

**Figure 3:**
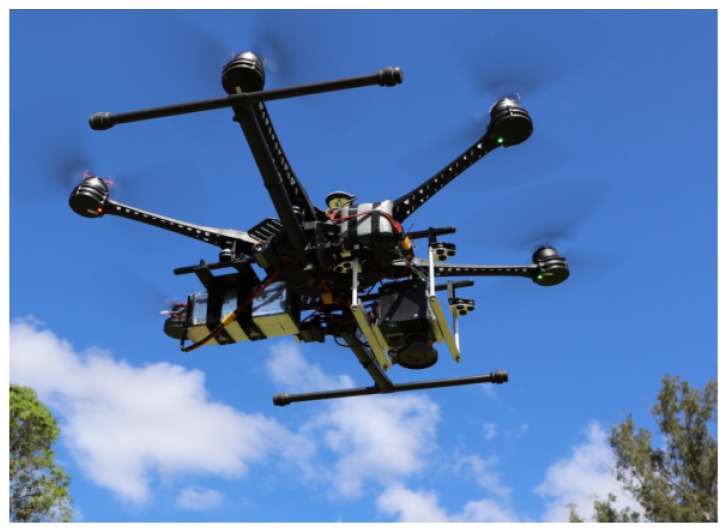
The DJI S800 EVO (DJI, Guangdong, China) unmanned aerial vehicle (UAV) flying in Cape Range National Park, Western Australia (WA), Australia.

A DJI S800 EVO Hexa-rotor UAV (DJI, Guangdong, China) was employed in the study area following a designed mission route with DJI Ground Station 4.0 software. As shown in Figure 3, the UAV featured high-performance brushless motors, a customised dampened gimbal providing active three-axis stabilisation of the sensor payload (levelled out to ensure the sensor was pointing permanently in the direction of the ground), a total weight of 3.9 kg, and dimensions of 1180 mm × 1000 mm × 500 mm. The flight mission was performed at an altitude of 66.9 ± 4.6 m, an overlap of 80%, side lap of 50%, and a route length of 6.6 km at 16.2 km/h. The horizontal and vertical GSD were approximately 1.0152 cmpixel in both cases.

### 2.5. Software

Various software solutions were used through the development of this research. In order to prepare the data, more than 500 raw images were filtered and orthorectified using Agisoft PhotoScan 1.2. With this software, an orthomosaic image of 44,800 × 17,200 pixels of 2.4 GB was generated. Due to the huge image size and possible random-access memory (RAM) limitations, this image was split into 4816 items of 400 × 400 pixels in Tagged Image File (TIF) and Keyhole Markup Language (KML) formats. A group of representative samples in cropped regions was extracted and subsequently labelled using GNU Image Manipulation Program (GIMP) 2.8.22 to fit the classifier. The generated image set was processed using Python 2.7.14 programming language and several third-party libraries for data manipulation and machine learning, including eXtreme Gradient Boosting (XGBoost) 0.6 [28], Scikit-learn 0.19.1 [29], OpenCV 3.3.0 [30], and Matplotlib 2.1.0 [31].

### 2.6. Data Labelling

**Figure 4:**
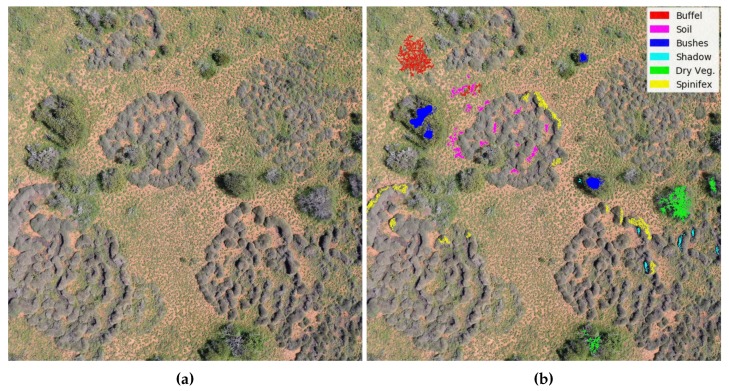
Image labelling. (**a**) Representative sample. (**b**) Highlighted regions using bright colours.

Due to the variety of conditions in which invasive grasses were found in Cape Range National Park, 10 images were selected and analysed using photo interpretation. Invasive grasses (buffel grass and spinifex), as well as common objects in the area, were highlighted using bright distinguishable colours as depicted in Figure 4. Regions were coloured through the “Bucket fill” tool of the GIMP software.

To perform image labelling, a mask for each image sample was generated by assigning integer values for every highlighted pixel. Each bright coloured pixel was filtered from every sample using Equation (1).

(1) $$H_{\left(x,y\right)}=\left\{\begin{array}{cc} a & \mathrm{if}\;S_{\left(x,y\right)}=F_{\left(R,G,B\right)}\\ 0 & \mathrm{otherwise} \end{array}\right.$$

where *H* is the mask for each sample *S* and *a* is the integer value for every bright colour value $F_{\left(R,G,B\right)}$. Values for *a* were set as follows: 1 = buffel grass; 2 = soil and road; 3 = bushes; 4 = shadow; 5 = dry vegetation (Dry Veg.); 6 = spinifex.

### 2.7. Classification Algorithm

Algorithm 1 was utilised for the training and prediction stages. It identifies and filters the highlighted regions mentioned in Section 2.6, trains a gradient boosted decision tree classifier, cross-validates the classification rates, predicts unlabelled data, and displays the results.

The training section of Algorithm 1 comprises several steps to load, preprocess the data, and fit an XGBoost classifier. The processing stage transforms the read data into an array of features or attributes, which are consequently processed by the classifier. As described in the algorithm, in order to obtain the feature array *D*, representative sample images *G* are firstly converted from their default RGB colour model into the hue, saturation, value (HSV) colour model in Step 3. Then, a set of filters are applied on *G* and their outputs inserted into *D* subsequently, as mentioned in Step 5. The two-dimensional (2D) filters calculate the variance into a subset of pixel neighbours contained in a window, following Equations (2) and (3).
**Algorithm 1** Detection and segmentation of invasive grasses using high-resolution RGB images.
*Required:* orthorectified image set *I*. Representative samples set *G*. Sample masks set *H*
**Training**1:**for**
i←1,n
**do**▹n= total images in *G* (labelled data)2:    Load $G_{i}$ and $H_{i}$ images3:    Convert colour space of $G_{i}$ into HSV4:    Insert each colour channel into a feature array *D*5:    Use 2D filters on $G_{i}$ and insert their outputs into *D*6:    From $G_{i}$ and $H_{i}$, filter only the pixels with assigned labelling on *D*7:**end for**8:Split *D* into training data $D_{T}$ and testing data $D_{E}$9:Create a XGBoost classifier *X* and fit it using $D_{T}$10:Use K-fold cross validation with $D_{E}$▹ number of folds = 1011:Perform grid search to tune *X* parameters
**Prediction**12:**for**
i←1,m
**do**▹m= total images in *I*13:    Load $I_{i}$ image14:    Convert colour space of $I_{i}$ into HSV15:    Scan every pixel and predict the object using *X*16:    $O_{i}\leftarrow$ Convert the data into a 2D image17:    Export $O_{i}$ into TIF format18:**end for**19:**return**
$O_{i}$

(2) $$X=\frac{1}{w^{2}}\left[\begin{array}{cccc} 1 & 1 & \cdots & 1\\ 1 & 1 & \cdots & 1\\ \vdots & \vdots & \ddots & \vdots \\ 1 & 1 & \cdots & 1 \end{array}\right]$$

(3) $$s^{2}=E\left[X^{2}\right]-{E[X]}^{2}$$

where *X* is the kernel of the filter to estimate the mean value of the processed image, *w* is the window size, and $s^{2}$ is the variance defined as the subtraction between the estimation of mean of square and the square of mean. Thus, the array of features *D* for this case study contains 10 items as follows: hue, saturation, value, variance filters on hue where *w* equals 3 and 15, variance filters on saturation where *w* equals 3 and 15, and variance filters of the grayscale image from $G_{i}$ where *w* equals 3, 7, and 15. Later, as described in Step 6, pixel locations that were previously labelled are filtered using masks *H*, following Equation (4).

(4) $$D_{j}=\left\{\begin{array}{cc} \left[G_{i}\left(x,y\right),\;H_{i}\left(x,y\right)\right] & \mathrm{if}\;H_{i}\left(x,y\right)\neq 0\\ \mathrm{null} & \mathrm{otherwise} \end{array}\right.$$

where $D_{j}$ is the 2D output array of the operation, $G_{i}\left(x,y\right)$ is the sample image, and $H_{i}\left(x,y\right)$ is the labelled counterpart of $G_{i}$ at position (x,y). In total, 342,626 pixel-wise samples were filtered and subsequently split randomly into a training (75%) and testing (25%) data array. In Step 9, data are processed into the XGBoost classifier, which is a state-of-art decision tree and gradient boosting based model created by Chen and Guestrin [28] that is optimised for large tree structures, high execution speed, and excellent performance. Hyper-parameters for this classifier such as the number of estimators, the learning rate, and maximum depth are estimated by running a grid search method in Step 11. This technique evaluates a combination of multiple values for each hyper-parameter, returning the optimal combination of those for the classifier. For this case study, the optimal hyper-parameter values to obtain an accuracy-robustness balance without causing over-fitting are:

estimators=100,learningrate=0.1,maximumdepth=3

where “estimators” is the number of trees, “learning rate” is the step size of each boosting step, and “maximum depth” is the maximum depth per tree that defines the complexity of the model. For the prediction stage (Steps 13–17), all the orthorectified images are processed in a loop using the trained classifier and the same data conversion considerations applied at the training stage. Finally, classified pixels for each image are painted in distinguishable colours and exported in TIF format, compatible with geographic information system (GIS) platforms.

## 3. Results

Segmented images for photo interpretation as well as accuracy indicators were implemented for validation purposes. In total, 85,657 labelled pixels were evaluated from the test data set $D_{E}$ to assess Algorithm 1. The confusion matrix of the classifier is presented in Table 1.

From 25,795 instances of pixels labelled as buffel grass, the algorithm predicted correctly 25,256 pixels and reported misclassifications of 362 pixels as spinifex, 156 pixels as bushes, 17 pixels as soil and 4 pixels as dry vegetation. Similarly, 25,196 pixels were successfully predicted as soil, with 17 misclassifications; 3913 pixels as bushes with 737 misclassifications; 7729 pixels as shadow with 1 misclassification; 5734 pixels as dry vegetation with 185 misclassifications; and 15,649 pixels as spinifex with 701 misclassifications. Based on these numbers, a classification report is generated as shown in Table 2.

Here, precision is the ratio between true positives and the sum of true positives and false positives, recall is the ratio between true positives and the sum of true positives and false negatives, f-score is the mean value between precision and recall, and support is the number of tested pixels per class. For this case study, precision errors indicate the output of misleading results by labelling wrong classes, whereas recall errors show the output of incomplete class detection.

Overall, the majority of the classes were successfully classified. For buffel grass and spinifex, most of the misclassified pixels were attributed to their counterpart class; these misclassification rates were small, representing for buffel grass precision and recall errors of 1.92% and 1.40%, respectively, and for spinifex values of 2.23% and 3.11%, respectively. Due to the high variation in greenness values of labelled buffel grass, specific areas of dry grass were classified as spinifex and vice versa. Similarly, misclassification of dry vegetation and spinifex instances (2.88% and 2.69%, 0.98% and 1.05%) occurred owing to many occurrences of this plant in senescence conditions. The classification rates were excellent for the shadow and soil classes, mainly due to the small variation in their visual properties such as their colour intensity, luminosity, and smooth texture. In contrast, the classification of the bushes class was not satisfactory at all, especially its recall rates, as indicated by a greater proportion of pixels classified as buffel grass (3.81% and 13.59%) and to a lesser degree, spinifex (0.49% and 1.74%).

**Figure 5:**
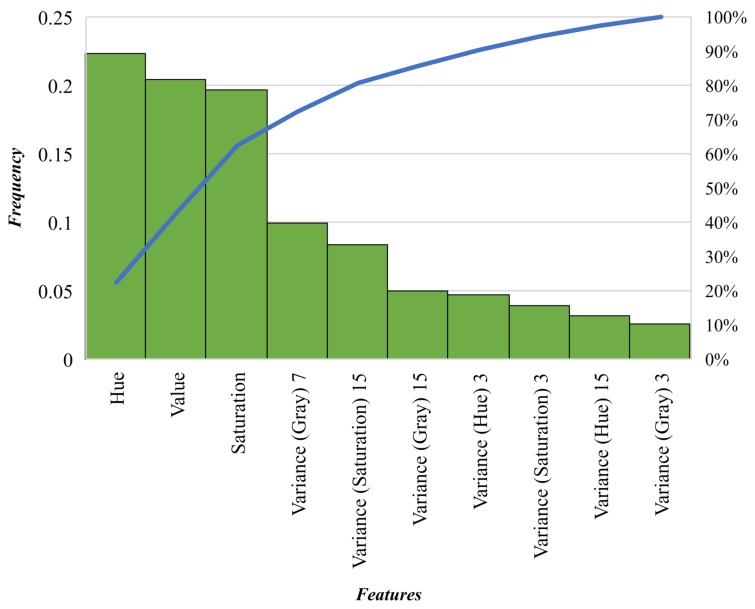
Relevance of each feature for the tuned classifier.

The proposed algorithm is capable of classifying invasive grasses and other vegetation with remarkable global precision rates of 97% and recall rates of 95.76%. The proposed method increases, nevertheless, the likelihood of classifying certain bush regions as buffel grass, with a recall rate of 84.15%. Considering an equal relevance of precision and recall for this investigation, the overall detection rate of the proposed method is 96.54%. The 10-fold cross-validation analysis achieved mean accuracy and standard deviation values of 97.54% and 0.042%, respectively. Furthermore, a feature’s relevance analysis was conducted for the XGBoost classifier. The embedded estimation function performs the sum of the instances each feature is split in its decision-tree-based structure. The importance of each feature is depicted in Figure 5.

**Figure 6:**
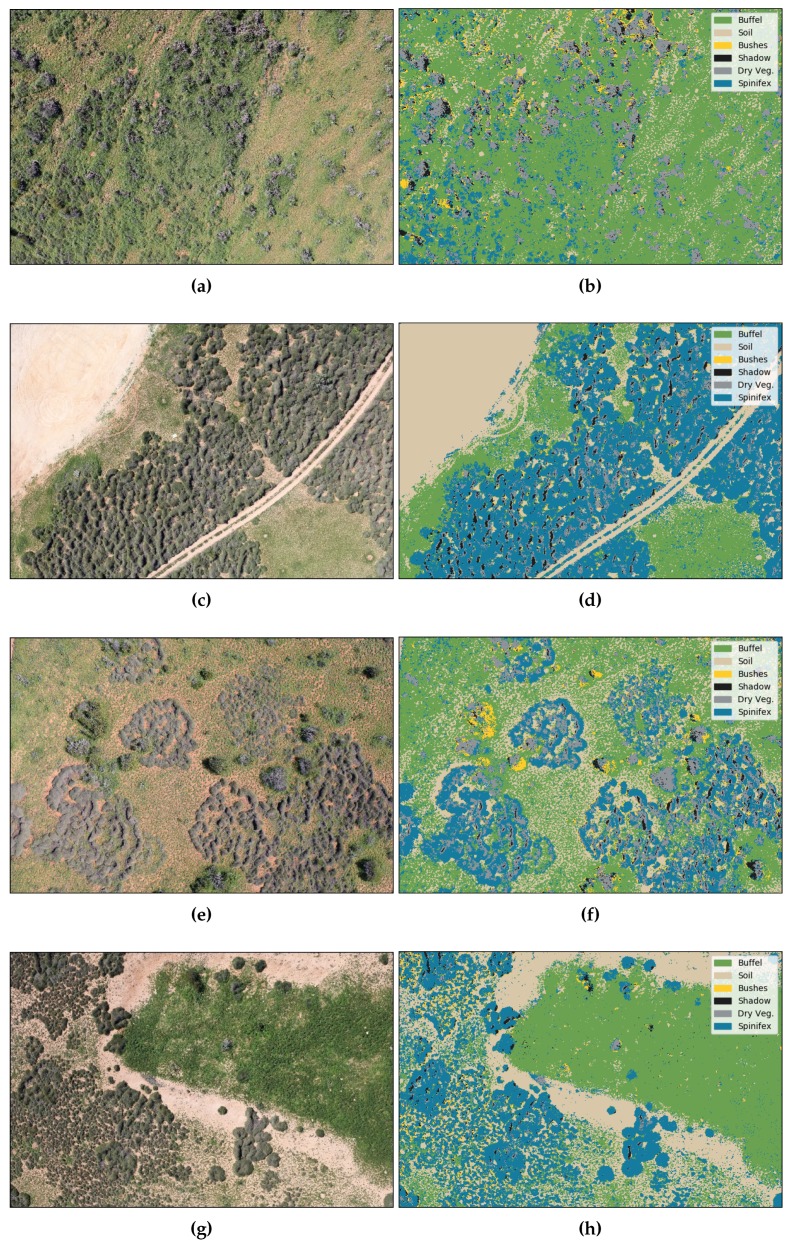
Pixel-wise segmentation from acquired red, green, blue (RGB) colour model images using Algorithm 1. (**a**, **c**, **e**, **g**) Orthorectified RGB images. (**b**, **d**, **f**, **h**) Final segmentation with predicted classes.

Where each bar item from the *x*-axis represents the relative frequency each feature has in the classifier. Here, hue, value, and saturation scores demonstrate the significant relevance the features had on the model, representing up to 65% of the total instances. These ratings are followed by 2D variance filter images such as the grayscale image with window size of 7 pixels, and the saturation image with a 15-pixel window size. The filters with substantially large window sizes showed clearly the importance of classifying accurately certain objects whose pixels are presented in a set of textures, such as bushes and spinifex. An illustration of the prediction and segmentation outputs is depicted in Figure 6.

**Figure 7:**
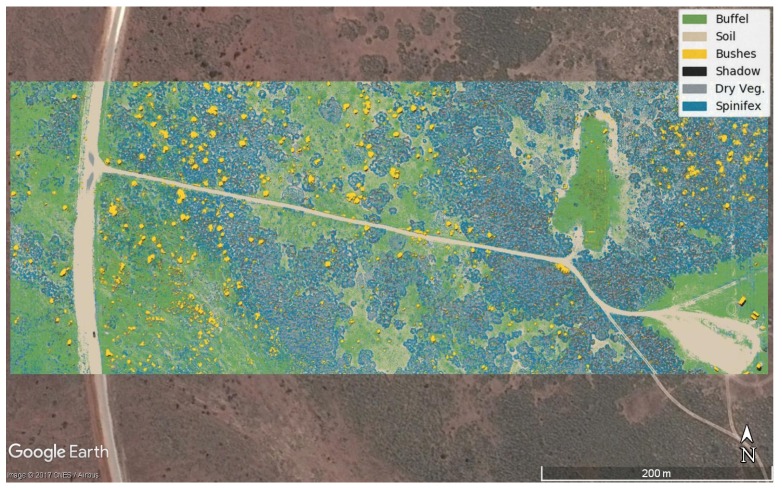
Prediction of invasive grasses in Cape Range National Park and its display in Google Earth.

Figure 6a,c,e,g depict representative samples where buffel grass, spinifex, bushes, soil, and dry vegetation are displayed at different densities and light conditions, whereas Figure 6b,d,f,h show the segmentation obtained from the proposed algorithm. As seen in the confusion matrix from Table 1, it is possible to obtain highly accurate segmentation results for the buffel grass, spinifex, soil and shadow classes. However, the segmentation results for the “bushes” class is unstable in some images and can be regarded in many cases as image noise. The segmented images can be loaded and displayed in any GIS software, as shown in Figure 7.

## 4. Discussion

Accuracy and segmentation indicators presented in Section 3 validate the proposed pipeline approach to map vegetation and invasive grasses in arid lands. Negligible proportions of observed misclassifications for “buffel” and “spinifex” classes may be attributed to human error during the labelling of sample data. That is strongly evidenced by evaluating the results for the “bushes” class where the number of misclassified pixels is attributed to a challenging image labelling task. These inaccuracies occurred because the visible colour properties of bushes from the RGB sensor showed many similarities with other vegetation. From environmental monitoring and biosecurity perspectives, the proposed method is capable of providing critical information such as the distribution of invasive grasses, density values of invasive species in arid lands, and estimation of their expansion values for the short and mid-term, among others.

The present study represents a competitive approach for the use of UAVs and machine learning-based classification models compared with alternative solutions. It complements the research outcomes on buffel grass of Marshall et al. [19] by confirming a feasible, accurate, lightweight and relatively cheap solution for invasive grass mapping. With regard to invasive grasses in arid lands, this paper has demonstrated that using only high-resolution RGB images and single pixel-wise classification satisfies the need for accurate and efficient detection and segmentation solutions.

It is noteworthy that the invasive grasses in this study had negligible size variation, background clutter, occlusion, and viewpoint variation, constituting, apparently, an advantage. As opposed to senescence conditions, varied levels of grass density and illumination variation did not represent additional challenges. However, acquired data is insufficient for performing further classification tests with changes in illumination in the study area, such as acquisition tasks at different times during the day and under cloudy conditions. These parameters might alter the detection rates of the presented approach, and further research should be conducted under these conditions. The processing of imagery with small GSD values demonstrates how UAV-based remote sensing equipment has improved sensing capabilities compared with satellite and manned aircraft for invasive grass assessments.

Future research should analyse the efficacy of supervised and unsupervised algorithms to label vegetation and specifically invasive grass species accurately, and integrate the best approaches in the proposed pipeline. Additionally, new efforts should be focused on improving the performance of the entire pipeline process as well as the aggregation and evaluation of unsupervised classification algorithms for image labelling tasks using RGB pictures only. Although the amount of previous research in optimising machine learning models is significant, specific areas might be improved for real-time applications, such as orthomosaic-based processes and a better software integration into a single solution.

## 5. Conclusions

This paper proposed an integrated pipeline methodology for mapping vegetation and invasive grasses in arid lands. The methods were demonstrated by mapping buffel grass and spinifex in remote areas of WA through the use of UAVs, high-resolution RGB imagery, and gradient boosted decision trees. The presented approach illustrates detection rates of 96.75% and 96.00% for single mapping of buffel grass and spinifex, respectively, and a multiclass detection rate of 96.54%. Invasive grasses were accurately detected at different spatial concentrations with a GSD of up to 1.015 cm/pixel, demonstrating how UAV data collection can be useful for invasive grass detection at early stages. This case study demonstrates the implementation of unmanned aerial systems and machine learning for a feasible, accurate, and lightweight assessment of invasive grasses in arid and semi-arid lands. Future work will focus on integrating unsupervised and supervised methods for vegetation data labelling in order to reduce processing times.

## Figures and Tables

**Table 1:** The eXtreme Gradient Boosting (XGBoost) classifier confusion matrix.

	Predicted	Buffel	Soil	Bushes	Shadow	Dry vegetation	Spinifex
**Labelled**	Buffel	**25,256**	17	156	0	4	362
	Soil	15	**25,196**	1	0	1	0
	Bushes	632	1	**3913**	2	21	81
	Shadow	0	1	0	**7729**	0	0
	Dry vegetation	8	10	6	2	**5734**	159
	Spinifex	508	2	20	0	171	**15,649**

**Table 2:** Classification report from confusion matrix of Table 1.

Class	Precision (%)	Recall (%)	F-Score (%)	Support
Buffel	95.60	97.91	96.75	25,795
Soil	99.88	99.93	99.90	25,213
Bushes	95.53	84.15	89.84	4650
Shadow	99.95	99.99	99.97	7730
Dry vegetation	96.68	96.87	96.78	5919
Spinifex	96.30	95.71	96.00	16,350
Mean	97.32	95.76	96.54	∑ = 85,657

## Supplementary Materials

Supplementary File 1

## Abbreviations

The following abbreviations are used in this manuscript:

2D	Two-dimensional
CMOS	Complementary metal–oxide–semiconductor
Dry Veg.	Dry vegetation
GEOBIA	Geographic Object-Based Image Analysis
GIMP	GNU Image Manipulation Program
GIS	Geographic information system
GPS	Global positioning system
GSD	Ground sampling distance
HSV	Hue, saturation, value colour model
KML	Keyhole Markup Language
MDPI	Multidisciplinary Digital Publishing Institute
RAM	Random-access memory
RGB	Red, green, blue colour model
TIF	Tagged Image File
UAV	Unmanned Aerial Vehicles
WA	Western Australia
XGBoost	eXtreme Gradient Boosting
